# Revised paper ASIM-D-21-00055R1: “Consulting properly rather than acting”: advocating for real patient involvement in summative OSCEs

**DOI:** 10.1186/s41077-022-00213-4

**Published:** 2022-06-06

**Authors:** Grainne P. Kearney, Jennifer L. Johnston, Nigel D. Hart, Kathy M. Cullen, Gerard J. Gormley

**Affiliations:** Centre for Medical Education, Queen’s University Belfast, Whitla Medical Building, 97 Lisburn Road, Belfast, Northern Ireland BT9 7BL UK

**Keywords:** Simulation, OSCES, SPs, Institutional ethnography

## Abstract

**Background:**

In this “Advancing simulation practice” article, we offer an expose of the involvement of real patients in Objective Structured Clinical Examinations (OSCEs), inviting educators who traditionally involve solely SPs in their summative OSCEs to consider the practice. The need for standardisation in summative assessments can make educators understandably wary to try this, even if the rhetoric to involve real patients is accepted. We offer this as an instance of the tussle between standardisation and validity experienced throughout health professions education.

**Main text:**

We offer our experience and empirical evidence of this simulation practice, based on an institutional ethnographic examination of the involvement of real patients in summative OSCEs from an undergraduate medical school in the UK. Our critique demonstrates the merits of this approach as an assessment environment closer to the real clinical environments where these soon-to-be doctors interact in a more authentic way with real patients and their illness experiences. We balance this against the extra work required for all involved and suggest the biggest challenge is in the reorientation work required for both Faculty and students who are institutionalised to expect standardisation above all in assessment.

**Conclusion:**

We advocate for involving real patients in summative OSCEs and hope that readers may feel compelled and empowered to foster this shift in mindset required to introduce this practice into their assessments.

## Introduction

Objective Structured Clinical Examinations, better known as OSCEs, have molded and morphed to secure their place as a ubiquitous form of assessment throughout health professions education (HPE). Drawing upon many cornerstones of simulation — including SP methodology (SPs referring interchangeably to simulated patients, standardised patients or simulated participants — hereafter referred to as SPs), concepts of scenario design and use of manikins — OSCEs are in effect constructed forms of reality that facilitate judgement on individual’s competencies. In the shift towards a competency-based model in HPE, the sustained emphasis on an outcomes-based approach to teaching and assessment [1] ensures OSCEs continue to dominate summative assessments internationally, albeit with variations in delivery.

In their AMEE guide, Khan et al. define OSCEs (through their consolidation of definitions in the literature) as “An assessment tool based on the principles of objectivity and standardisation” [2]. Traditionally, OSCEs involve patient roles being played by SPs, and authors have previously advocated that SPs should be actively involved in the co-construction of simulation scenarios depicting consultations [3], common practice described in the literature [4, 5]. Work with SPs in OSCEs is within the expressed spirit of commitment to standardisation. This discussion feeds in to a wider but prevailing criticism of OSCEs around perceived lack of authenticity [6], a feeling of being far removed from real clinical practice and patients. Many have warned of the potential unintended outcomes of highly simulated set ups [7, 8]. Bearman and Ajjawi [9] described exclusion of real patients from OSCEs but a move is growing momentum where real patients can be involved in the co-creation of learning materials [10].

The purpose of this article is to offer our experience and empirical evidence on the involvement of real patients in summative OSCEs in an undergraduate medical school in the UK, the practical application of which we hope is transferrable internationally. For clarity, we use the clumsy terminology of “real” patients to distinguish from SPs. This is not to say that SPs cannot also be patients in their own right but on the day of an OSCE, they take on a role that they have been trained for and briefed in, as an increasingly professionalised group [11, 12]. We will come back to this distinction in more detail later in this article. More broadly, we detail an instance of the ongoing tussle health professions educators face on a daily basis, balancing a need to deliver standardisation against a desire for authenticity, and offer readers a description of the work involved in striving towards some real patient involvement. An institutional ethnographic examination is presented here detailing the merits of this approach, tampered alongside the challenges it brings, with the intention of encouraging a change in mindset to normative OSCE practices.

## Background

The basis of this “Advancing simulation practice” article is derived from a study which used institutional ethnography [13–15] as the approach to inquiry to critically examine OSCE practices. Institutional ethnography (IE) is a complex, critical qualitative theory/methodology, conceived by Dorothy Smith drawing on her reading of Marx’s materialism and her experiences in the feminist movement. The focus in the IE approach is on what people actually do on the ground as their “work”; it then moves to investigate where this work is organised from in a governing sense — the “institution”. With roots in activism and social justice issues, it has been widely used to study health care settings but is gaining momentum in HPE (see [16] for more detail on this approach and its potential applications in HPE). When using IE, researchers reflexively declare the standpoint that they are taking in the study at the outset.

In the early stages of an IE study, it is not known which threads of inquiry the researcher will take up. Whilst the crux of this research was a problematisation of the dominance of standardisation in OSCE practices and how this traces back to the overruling demand for accountability, a vibrant and unexpected thread that developed through the study was a critique of the involvement of real patients in summative OSCEs.

GK, an academic General Practitioner (GP), spent the academic year 17/18 collecting data on summative OSCEs (the final clinical assessment prior to students graduating) in the medical school where she worked. KC is the Academic Lead for Assessment of Final year students with a leadership role in planning, delivering and reviewing OSCEs involving both SPs and real patients. GG, JJ and NH are all Academic GPs in this medical school who, along with GK, regularly examine in OSCEs involving both real patients and SPs. The medical school is a large undergraduate school in the United Kingdom (UK); these summative OSCEs take place 6 months before the students qualify and start work as doctors. The estimate of the total number of people involved in these summative OSCEs is 660 people, including 253 students and approximately 75 SPs, 80 real patients, 160 examiners and 90 Faculty.

These summative OSCEs took place over 3 days, with sixteen stations in total (six involving real patients — explained below). Each station was stand alone, lasting 8 min with a specific objective such as history taking, examination or procedure demonstration. Stations were developed by the wider OSCE team, including Clinical Academics (medical doctors who hold a joint clinical and academic role in the medical school) and administration staff. With the help of a statistician, they quality assured the assessment including post hoc psychometric analysis. SPs attended a training session in advance of the OSCEs for briefing and practice of the station, led by one of the Clinical Academics. There is a further briefing on the day, and they are invited to feedback on their station afterwards. Real patients were prepared in an individual and informal way by one of the Clinical Academics on the day. The real patients interacted with a smaller number of students, typically up to eight. Examiners undergo training when first signing up to be an examiner followed by two yearly updates.

Data collection in this study involved many hours of ethnographic observation of the work involved in OSCEs, observing the team that planned, delivered and later reviewed the OSCEs as well as the work of the students, newly qualified doctors, examiners and SPs involved in these OSCEs. Observations were recorded as fieldnotes; for example, there were 32 h of observation during the OSCEs themselves. Alongside this, the researcher interviewed these people both formally and spoke to them more informally about what they were doing and how they knew to do it (17 interviews in total). In addition, the research team analysed the texts identified by participants during observations and interviews as texts they used in their OSCE work, these included texts produced within the medical school such as mark sheets and texts more widely available, such as from the regulatory body. Data collection and analysis were iterative, focused on what people did as their work and how they knew to do it, i.e. what roles texts played in their work. The study had full ethical approval (Ref: 17. 29v2).

To put this article in context, we now describe the trajectory of involvement of real patients in Final year summative assessments in this medical school, as explained to GK by long serving academic and administrative staff. Up until the early 2000s, the clinical part of Final year assessments involved “Long cases” (where students spent 40 min with a patient completing the taking of a full history and examination and preparing to present their findings to two examiners) and “Short cases” (where students were taken by two examiners to a variety of patients to illicit clinical signs and suggest “spot diagnoses”). These patients were mostly current inpatients asked by the ward doctors to be involved, expertly overseen by Senior Nurses. Involvement of inpatients became less feasible due to a number of challenges being felt internationally; fewer patients were being admitted for inpatient management, those admitted were more acutely unwell and wards were increasingly specialised. The final straw came when hospital management raised concerns about bringing these patients together, due to infection control worries. Outside this specific medical school, concerns expressed about perceived variability for students in Long cases [17], in combination with a desire for more objective clinical assessments set the scene for the introduction of OSCEs from the 1970s [18, 19]. So, with these on the ground problems, coupled with the shift in the medical education literature, OSCEs were introduced in this medical school in the early 2000s with a widening team of administrative and managerial colleagues including a psychometrician supporting Clinical Academics in organising the assessments. To put this into an international context, this move to OSCEs was reflected in many medical schools internationally at the end of the twentieth century. The majority of medical schools introducing OSCEs around this time in the UK replaced real patients with SPs, but this medical school deliberatively continued to involve a small number of real patients in their summative OSCEs as a hybrid model alongside SPs, under the leadership of Clinical Academics. Ongoing real patient involvement mostly encompassed “signs” that students could examine, but in the year that this study took place, a decision was taken (unrelated to the research) to invite real patients to also take part in history-taking stations.

## Critical comparison of real patients and SPs in OSCEs

In this section, we present composite accounts of interactions of students with SPs, and for comparison, interactions with real patients in OSCEs. Composite accounts are “accounts constructed by the researcher that are built from the corpus of data collected (e.g., interviews, observations, and texts)” [20]. These composite accounts are based on GK’s ethnographic data, from her field notes taken whilst observing during the OSCEs combined with interviewees’ experience of being involved in OSCEs as Faculty, examiners and students. Whilst they are presented here almost as transcripts, some editing has been applied for them to work in this format.

**Fig. 1:**
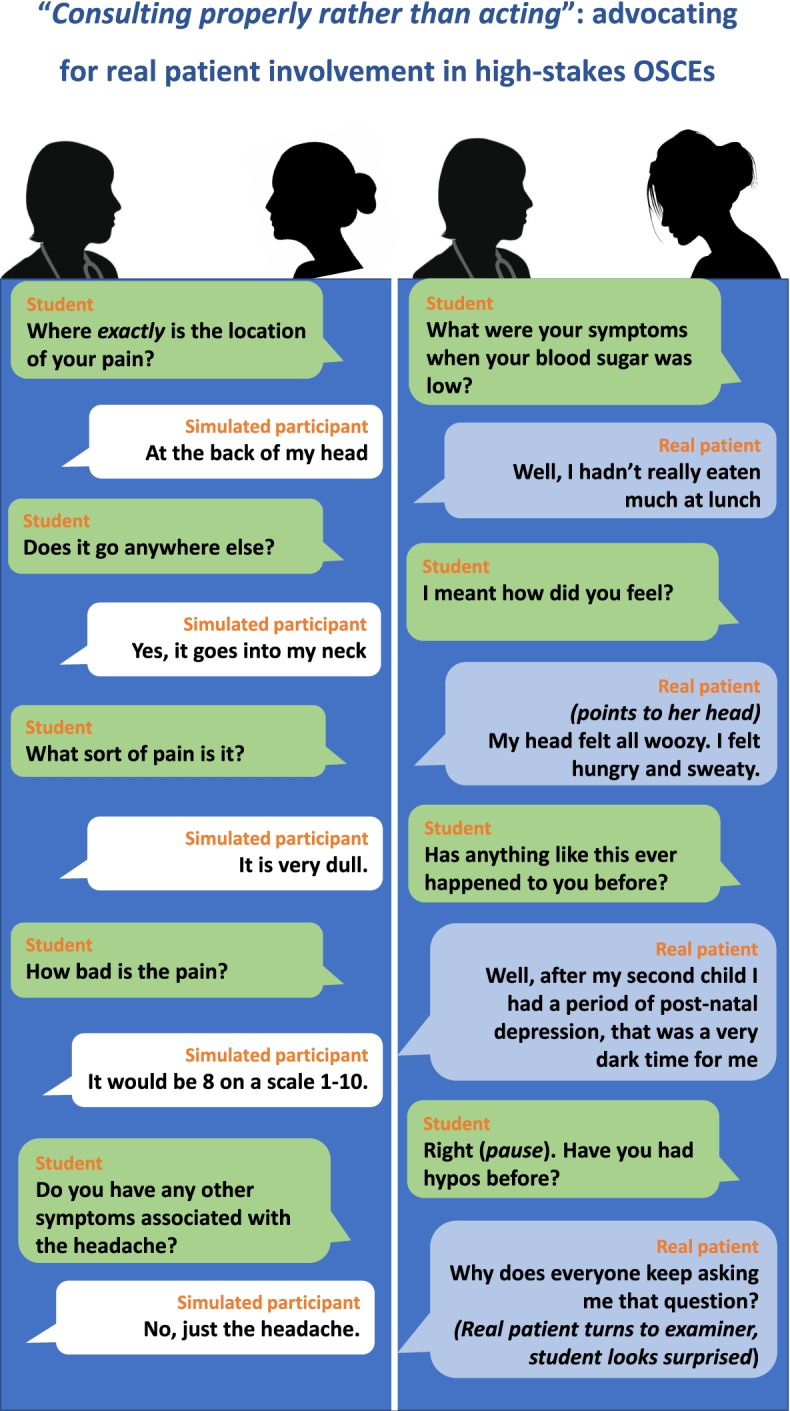
Composite accounts of interactions between students and SPs and between students and real patients

At the outset and in keeping with the tenets of IE, it is important to state that the critique here is not aimed at the students, SPs, examiners or team in charge of OSCEs, all doing their best within the confines of their work. It is instead on how their work processes are organised, how imposed recognition of process plays out in their work and the consequences of this (Fig. 1).

In the interaction between the student and the SP, the SP has come to expect the checklist of questions that the student will ask, through their experience in teaching, previous OSCEs and their training. They answer the questions as asked without giving away any information that the student has not specifically sought. Their highly edited scripts are often single symptom focused, quite a contrast to consultations with real patient whose real-life scripts contain many symptoms which may or may not appear to be linked in a clinician’s mind. As exemplified above, when asked about the “location” of the pain, the SP is trained to give a specific spot where pain has originated. They do not at this point reveal a secondary location, where they have been trained to report (only if asked) that the pain radiates to which allows the student to secure the relevant mark for “radiation”. Through their training, SPs have come to understand these clinical concepts such as location and radiation of pain. A real patient might have described how they feel pain both at the back of their head and at the back of their neck; their thinking has not been organised to consider their pain in terms of location and radiation.

The SPs’ training and experience introduce them to other medical terminology taught to students with regard for example to the “character” of the pain such as “dull” and they will be very aware of conventions such as scoring pain from 1 to 10. Real patients may or may not have come across such jargon. A student said in an interview, “I think kind of when you’ve come to Final year, the simulated patients, you almost know a lot of them, you’ve seen them before and you almost get to know how they give a history, does that make sense?” This student recognises that the SPs have been trained in the same discourse as they have as medical students, reflecting Bleakley and Bligh [21], who state that “For the student, the patient’s language is mediated through medicalized language shared with the doctor, and the patient’s experience is then sub-ordinated to this technical language (and, paradoxically both reduced and simplified for instrumental purposes).” We will later discuss how different training for the SPs will not entirely address these concerns.

The other composite account from this study details the interaction between the student and a real patient, where the real patient has not been trained for their role in OSCEs. Their illness experience narrative may not always fit well with the checklist of questions the student is armed with or that the examiner has in front of them (the mark sheet). In this interaction, the real patients may be more likely to use gesturing etc. to describe their lived experience. They may not use jargon or understand it when used by the student. They may coin terms they feel best describes how things felt for them personally which may not be on the list of pain “characters” the student has been taught. The real patient offers extra details of how they felt that were not specifically asked for — “I then felt hungry and sweaty”. One of the Clinical Academics stated their worry that when a real patient gave their own histories, they might give away “too much information”. The real patients can give information that the students may consider irrelevant, for example, the students will not link this patient’s low blood sugar with post-natal depression, despite the patient linking them. This is an example of complexity which so often inhabits real practice, but which does not sit easily in OSCEs, where the focus is more one dimensional, with one specific complaint that a student is taught to ask for and explore. The response of the real patient to the question is reflective of how a patient may turn to the most senior doctor present when they have questions about the interaction for reassurance, as the clinician with the most experience. The potentially less predictable response of a real patient increases the risk of threats to the psychological safety of the student. We will return to this point.

The Clinical Academics in this institution maintained how they did not and would not train the real patients or give them what they referred to as a “pretend history” to describe, instead inviting them to answer questions in a way that was comfortable for them. Students revealed in the interview their surprise when one of the real patients did not know their medication list in the OSCE; this student described how they expected that the real patient would be more “rehearsed”. Other students reported that they felt the real patients had not been properly prepared for the assessment. In contrast to the student quoted above, who recognised that the SPs’ training reflected their teaching as medical students, we believe the comments of students on perceived lack of preparedness on the part of the real patients reflect that students also expect real patients to speak and behave like SPs. In Sinclair’s famous ethnography of medical students, he stated that the students’ narratives when summarising patients’ histories were “a reconstruction of the patient’s experience as if the patient was a doctor” [22]. We consider the SP scripted histories as a reflection of a doctor’s representation of a patient’s disease (written through their experience of listening to patients) rather than a patient’s lived experience of their illness. These accounts compare and contrast a standardised biomedical model of disease, with illness experience which is embodied and contextual. The authors note this research took place at a time of growing evidence into real patients co-creating SP scripts [10], in order to bring this embodied experience and will align these two approaches in the conclusion.

### What can real patients bring to the OSCE assessment?

This is the “so what” of this “Advanced simulation practice” article, the reason to justify all the extra work for the organisers that we detail below. Real patients bring an experiential, lived perspective beyond that of a disease: their lived actuality of illness which Mol defines as “a patient’s interpretation of his or her disease, the feelings that accompany it, the life events it turns into” [23]. On asking the Clinical Academics in the interview why they chose to include real patients in the assessments, they spoke enthusiastically of “real validity”, where “authenticity is to be valued”. They espoused that real patients do not read textbooks and that they “present as they present” and tell it “as it was, just the way it was”. Real patients, in their unscripted way, described their illness in the way that they personally make sense of it. Their stories develop the rich tapestry of uncertainty and non-standardised messiness that clinicians learn from and work in.

Students spoke at length about how consulting with real patients in their assessments felt “natural” or “normal”. As one student explained, “it is such a natural chat compared to with an actor”. An SP also commented on this, stating about students in OSCEs, “I don’t know how much they see it as the genuine article, they seem to perform the role very well.” This idea of OSCEs as a performance has been well documented in the literature [6]. A different SP also picked up on this, describing student’s behaviour in OSCEs as “robotic” towards them, “I think a lot of them … they learn this mode, they go into the checklist and forget that they have got their patient”. This SP’s comment implies they feel objectified in medical education, also previously described in the literature [3].

Students talked of feeling more relaxed with real patients, describing reactive dialogue which contrasted with their consultations with SPs where they admitted to thinking mostly about what their next question would be. One student described how they adjusted their usual OSCE tactic of trying to ask as many questions as quickly as possible as they “worried that these [real] patients have given up their afternoon” and might find such common OSCE practices “strange”. Students admitted how they set aside their usual worries in OSCEs of appearing “too nice and too familiar” which they equated with appearing as if they were stalling. They likened their interactions with real patients in OSCEs to being “on a ward, you’re like, ‘how are things and how are you today?’ And you chat to patients to make them feel more comfortable.” Whilst the students talked of making the real patients feel comfortable, they inadvertently described their own increased ease within the assessment. Students and real patients laughed (genuinely) together at times in these assessments, on a day where more negative student emotions tended to be more notable. Students contrasted this with their interactions with the SPs, “so even though the actors are good, it’s not representative of how you would conduct yourself on the ward maybe”. OSCEs with real patients allowed students to demonstrate more closely how they would be in the workplace, as demonstrated in this quotation where a student stated that “It’s not an OSCE when it’s a real patient”.

Examiners observing the students interacting with real patients in these assessments also used the words “normal” and “natural” to describe the atmosphere. For instance, one examiner described the interaction as a “normal consultation. I could actually see them consulting properly rather than acting in an exam situation”. In this study, all examiners are clinicians, so like the student they sought to compare and contrast what they observed to their experience of clinical practice. The examiners talked of observing what they considered to be real empathy between the student and the real patient, as the student tried to understand the actualities of the experiences of the person in front of them. One examiner contrasted this to what they often considered in OSCEs, that students were “acting empathy”. This echoes some of the concerns that Hanna and Fin eloquently described as “simulation doctors … who act out a good relationship to their patients but have no authentic connection with them.” [24]. The clinical experience of the examiners allowed them to expand on their positive observations. They witnessed the students having to dynamically adapt their consultation skills with real patients in a way that might be necessary in real practice. As one examiner explained, “there is always a bit of adaptation which is what you have to do when there is a patient in front of you in real life. So, I think they are much more realistic … ”. The real patients bring a sense of normalcy and realness which promotes the development of a genuine connection with the student akin to what might happen in a clinical environment; as an examiner concluded, “nobody is acting but the relationship in that moment is real.”

It could be argued that this connection with patients, the relationship building, will happen organically when the students start to work as doctors and indeed will already be happening as they learn in clinical environments. We feel that the educational value of these senior students being assessed working with real patients is that it keeps their learning in context and focuses their minds towards their role as person-centred clinicians, helping them feel more prepared to work in clinical environments in the very near future.

Fundamentally, what real patients can bring to OSCEs is of course some authenticity. As stated in the introduction, the apparent lack of authenticity in OSCEs is a well-documented tension those in charge of OSCE delivery and simulation-based assessments have to grapple with continuously.

### Why is it different with real patients?

We considered why the OSCEs with real patients were different to those involving SPs who are obviously also “real” people and who may have patient experience themselves. At the surface level, there are obvious differences in how they are recruited. The real patients are asked at the request of specialists involved in their care and are not remunerated for their time (though are given a gift voucher). Some reported to one of the Clinical Academics that they saw it as their opportunity to give back. In contrast, the SPs have put themselves forward to interview for formal recruitment for involvement in teaching and assessment in a way that fulfils the employment requirements of the University and they are paid for their time. In this medical school, the SPs are predominantly over the age of 60, often retired and mostly white. They self-select as people who feel that they have the necessary skills and ability to articulate themselves well in this work. Whether or not they have experience themselves of being real patients, they are not employed in that role. In contrast, the real patients will come from a variety of backgrounds and have a variety of different levels of education. This more random approach to real patient recruitment may result in this group being closer to the variety of patients that the students will encounter in real clinical practice.

Perhaps more important is what happens after recruitment. SPs, in their work, receive outlining scripts and specific training in their roles; their training involves inadvertently learning some medical knowledge relevant to that role. Carrying out this role repeatedly with students, they come to know what questions students will ask and how students approach examination. Fundamentally, real patients very deliberately in this medical school did not receive training for their involvement on the assessment days. The Clinical Academics repeatedly stated how the real patients would not be scripted, would not be acting and would use their own names in the stations. We argue that it is the non-institutionalisation or non-professionalisation of these real patients and their lack of training and therefore of standardisation that allows their social knowledge of illness to endure and invites students to form genuine relationships with their humanity; real patients remain in the patient role. By non-professionalisation, we are not implying any lack of knowledge or education on the part of these real patients. Rather their experiential social knowledge of their illness, which they have molded, and which has molded them, is different and unique for each individual; they are the experts in their individual lives and illnesses. Their narrative is not dictated to them in a way that privileges the “reconstruction of the patient’s experience as if the patient were a doctor” quoted from Sinclair above [22]. It is conventional not to involve SPs in OSCE stations with some relevance for their personal declared medical conditions due to psychological safety where it has the potential to be traumatising for the SP [25, 26]. It could also be argued that this would stand in the way of standardised portrayal across different SPs involved in this station. This active separation away from any real patient experience that many of SPs have when taking part in OSCEs was recognised by a student who explained, “Once you’ve become a simulated patient, you’re not a patient. Like you’ve been trained to do the role and it’s a very different role to that of a patient … ”

A newly qualified doctor in the interview gave a tongue-in-cheek explanation of how they saw this difference playing out in their experience as a student and now a clinician.

The example that I gave … was like a neurological exam. In an OSCE you could do a perfect, to the letter neurology exam, tick all the boxes. But when it comes to real life, and you are talking to someone who is not a simulated patient, because to my mind, every “neurology” patient (gestures neurology in quotation marks) was a simulated patient, whether you like it or not. Regardless of how standardised it is, they are sort of preconditioned to ‘oh lift your leg up for me’ and they know what to do. But if you are talking to a real patient, who is a little bit deaf and a little bit confused, who doesn’t really like doctors and would rather get home to the cows. And you are trying to examine dysdiadochokinesis and he’s like ‘what are you on about?’ So, you can do a perfect neurologic exam, but if you can’t communicate how to do it, you are not going to be able to do it.

Fundamentally, what we found different about real patients is in how they describe their individual social knowledge of their personal illness. Whilst SPs may or could be real patients, in the context of an OSCE, once they are loosely scripted, trained, rehearsed and standardised in the way of the institution, they no longer draw on their social knowledge of illness. Real patients, through their random recruitment, lack of scripting, lack of training and lack of standardisation, allow students to understand the sensuous actualities of living with illness rather having a biomedical disease. Later in this article, we offer a compromise position.

### The extra work with involving real patients

We hope we have made a strong case for involving real patients in OSCEs. However, in describing the involvement of real patients in OSCEs in this medical school, it is important to note that it was in only in less than half of the stations (six out of sixteen in the year data was collected). Whilst many of those involved in organising OSCEs cited the involvement of real patients as rewarding in the interview, they talked at length of the extra work, time and effort required to make it possible. Previous studies have detailed the work or labour involved in the general organisation of OSCEs [27]. In this institutional ethnographic study, we detail the work of involving real patients in OSCEs, revealing how much of this additional work pivoted on the perceived challenges to standardisation when involving real patients for all invested in these assessments. Therefore, a pragmatic “Advanced simulation practice” article on this practice necessitates description of this extra work and how this medical school managed it. In IE terms, this work is considered to be unseen, not because it is not sanctioned within the medical school but because the time and effort involved are not represented in documentation around OSCEs.

For those tasked with preparing for the OSCEs, the initial planning of stations involving real patients took substantially more time; roughly five times more time in the later meetings was spent planning the stations involving real patients as compared to those with SPs. Real patients were not actively involved in station writing in advance but brought their experience to bear in their answers on the day. The mark sheets were written more broadly to reward students for learning the broad details that would be relevant during the particular consultation rather than looking for very specific answers that two patients with the same condition may not both give. In addition, a substantial amount of time was involved in finding and securing the involvement of these real patients, especially in the numbers required for a large student cohort. This is in contrast to a single group email to ask for SP involvement. Those organising the OSCEs knew from experience not to contact the real patients too far in advance of the OSCE dates in case their condition might deteriorate or resolve (either through treatment or through the passage of time) in a way that would render their involvement of questionable value or not possible; all of these situations had happened in previous years. Even with this large amount of preparatory work, every year there are last-minute cancellations and no-shows. The team plan for between 33 and 50% extra real patients on the day, just as they do with the examiners.

Described so far are the mechanics of preparing for an assessment day where some real patients are involved; this is only part of the unseen work. Time, energy and patience are also required in preparing Faculty, students and examiners for this part of the assessment; the details of which are described below. Involving real patients requires reassurance for all who have been organised to value standardisation in OSCEs, above all. A shift away from seeing standardisation as the sole guiding principle of the assessment, towards the veneration of the illness experience brought by real patients, is necessary for all involved to feel comfortable and confident. This will not work without the commitment of all those involved.

Considering the specific needs of the students first, during the study, students regularly spoke of how they valued having real patients in their OSCEs but admitted in their next breath how they felt challenged by the seeming lack of standardisation in their interactions with them. One student exemplified this saying “I think I’d like to see more of it, as I think it is more representative of what you are going to be doing day to day as a doctor but I can understand why people maybe think it is not as good of an idea because there is variation. And after the OSCEs they were stories of ‘my patient didn’t tell me this’, or the signs are slightly different and obviously the histories are different between real patients. So, I see that it is harder to standardise.” They described how (other) students who want to take the “perfect history” might find an encounter with a real patient in an OSCE difficult, speculating how they might struggle with perceived “irrelevant” lines of questioning or where the student cannot “control” this history. Faculty gave examples from their experience where students got visibly frustrated during OSCEs with real patients and distracted by their non-standardised presentation.

In a similar vein, examiners, especially those who are not Faculty, will also need reassurance and guidance for their involvement. They should be encouraged to draw on their own clinical experiences with patients, and so rather than reward students parroting off lists of symptoms that may fall under a particular “system” to have faith in a more flexible style of mark sheet that will reward the dynamic clinical history taking skills required to consult with a real patient. Building such flexibility into mark sheets involves more of a domain-based style rather than a strict checklist, being less prescriptive of specific questions that students “must” ask for example and rewarding instead a comprehensive history where the questions were instead based on what the real patient was saying and how they were saying it. This slight shift in marking will require specific instructions for examiners and training. Faculty and examiners can be reassured that these stations involving real patients undergo the same psychometric interrogation as those with SPs (and indeed the post hoc psychometric analysis on this assessment was the same as in previous years).

We saw exemplified in figure that the non-standardised and non-institutionalised real patients may act or speak less predictably than a SP would, in a way that reflects what happens in real practice. Examiners will need training to ensure that the psychological safety of the student is maintained at all times. This may be an argument for ensuring that only senior students with much clinical experience encounter real patients in OSCEs.

The authors note that running OSCEs with SPs alongside real patients is the norm in this medical school — Faculty, examiners, students and the SPs have been organised to expect this set up and their work encompasses this hybrid model. We surmise if other institutions were to adopt this hybrid approach to OSCEs, further work would be required to prepare all these groups for this within a single assessment — students to expect different interactions from SPs than real patients within an OSCE session, Faculty when standing setting the OSCE, etc.

What we have described is not only the technical aspects of involving real patients but just as importantly, the extra work required in preparing the mindsets of all involved. In the UK, the General Medical Council (GMC), the regulatory body for doctors, are due to commence a National Licensing Examination in 2024 [28] for all doctors registering to work in the UK. The clinical side of this assessment is planned to be an OSCE or OSCE-like assessment, where individual medical schools will run their traditional summative OSCEs, providing evidence of how they meet the new regulations of the GMC [29]. As the GMC set their guidance, they are advocating for inclusion of real patient experience, something likely to present challenge for individual UK medical schools. The current guidance offered by the GMC mandates evidence from each medical school on how they recruit, train, brief and calibrate any SP or real patient involvement in future assessment [29]. We are aware that what we are promoting, involving some real patients in summative OSCEs, will substantially add to this workload as medical schools aim to ensure their processes satisfy the regulator wherever they are based and require a shift in thinking from the ground up, for all involved in OSCEs.

It is important to note participants in this study will have known the research team which may give rise to concern about how this power differential may have played out in data collection. However, the IE approach looks at the organisation of work and work processes, reflexively recognising the role that the researchers themselves play in data collection and analysis.

### The compromise

Advocates of SP methodology might question the thread of this article, pointing to the abundant literature which describes the many merits of working with SPs in OSCEs. There may be the view that the methodology, when rigorously implemented, with a different approach to SP training may mitigate somewhat the earlier student’s assertion that students “get to know how [SPs] give a history”; that SP methodology can provide a robust and unequalled mechanism by which to assure representative illness diversity with trans-curricular relevance; the inference being that the use of real patients with all their diversity diminishes those assurances. A tenet of an argument to contest this position would be that the methodology even when optimally realised can never fully capture the authentic expression of the lived illness experience. There are limits to the SP methodology just as there were with former exam formats (described previously) which only involved real patients. We as authors recognise the important contribution to the assessment made by SPs; what we are suggesting here is a compromise position working with both SPs and real patients but under very different social organisation, in order to embrace both realism and relevance, narrowing the gap a little more between simulation and practice.

### A note of caution

OSCEs are a constructed phenomenon. We not only have the ability to change their destiny but perhaps even a moral obligation to address their shortcomings. Would the public expect student doctors to be certified on their interactions with real patients before they are granted a provisional license to treat them? Some regulatory bodies are encouraging involving real patients [29] and their illness experiences in OSCEs; however, we urge some caution. In times of increasing regulation throughout HPE, we need to be careful that any potential to make real patient involvement in OSCEs mandatory or any attempt to standardise real patients in OSCEs may defeat the individualism and realism that we have celebrated here. With echoes of the historical concerns described above of clinical managers around cross-infection risk in assessment, we must be careful that another wave of change in the form of increased regulation does not cause further “cleansing” or sterilisation of how we deem medical students ready for the real world. This concern can be extended to real patients repeatedly being involved in OSCEs, could they equally become organised by a need to standardise and in time become a human role player themselves, a type of SP? We must think carefully about how we involve real patients at all possible points and remain true to their authentic voices, rather than succumb to the drive for their standardisation and professionalisation.

## Conclusion

The stated purpose in our introduction of this “Advanced simulation practice” article was to offer what we have learned through study and experience of involving real patients in summative OSCEs. We conclude involving real patients achieves a more naturalised and authentic assessment environment, closer to the real clinical environments for these soon-to-be doctors. These consultations with real patients demonstrate in a more genuine way how senior students interact and develop rapport with people rather than with checklists of symptoms, how they take the vast biomedical knowledge developed through their years in medical school and make it work for the individual in front of them. The non-standardised and non-institutionalised presence of real patients, still in the role as patients, helps students demonstrate if they are real-world ready, prepared for the unscripted messiness and uncertainty of the real clinical practice they are currently learning in and are about to work in. The examiner quoted in the title of this article stated, “I could actually see them consulting properly rather than acting in an exam situation”: for them, the presence of a real patient allowed for an authentic consultation moment, albeit in the surroundings of a standardised assessment.

Involving real patients in summative OSCEs requires much more work than organising their attendance on the day. Appetite already exists among the simulation community for the involvement of real patients in learning environments where they co-create SP scripts to make them more authentic or are involved in coaching SPs, or review of scenarios (10). We see these co-created scripts, delivered by SPs, to be the mainstay for more junior students in assessment but advocate alongside this practice for real patients themselves to deliver their own “scripts” in summative assessments for senior students. The positive disruption real patients can bring to highly standardised assessments necessitates a major shift in thinking for those involved in OSCEs, a reorganisation of what they consider to be a success in assessment. The work involves changing the heart and minds, but we advocate for finding ways to give it a go!

## Data Availability

Not applicable
